# Comprehensive Analysis of Rare Variants of 101 Autism-Linked Genes in a Hungarian Cohort of Autism Spectrum Disorder Patients

**DOI:** 10.3389/fgene.2019.00434

**Published:** 2019-05-08

**Authors:** Péter Balicza, Noémi Ágnes Varga, Bence Bolgár, Klára Pentelényi, Renáta Bencsik, Anikó Gál, András Gézsi, Csilla Prekop, Viktor Molnár, Mária Judit Molnár

**Affiliations:** ^1^Institute of Genomic Medicine and Rare Disorders, Semmelweis University, Budapest, Hungary; ^2^Faculty of Electrical Engineering and Informatics, Budapest University of Technology and Economics, Budapest, Hungary; ^3^Vadaskert Foundation for Children’s Mental Health, Budapest, Hungary

**Keywords:** autism spectrum disorder, rare variant, next generation sequencing, panel sequencing, rare variant burden, cluster analysis, syndromic autism

## Abstract

**Background:**

Autism spectrum disorder (ASD) is genetically and phenotypically heterogeneous. Former genetic studies suggested that both common and rare genetic variants play a role in the etiology. In this study, we aimed to analyze rare variants detected by next generation sequencing (NGS) in an autism cohort from Hungary.

**Methods:**

We investigated the yield of NGS panel sequencing of an unselected ASD cohort (*N* = 174 ) for the detection of ASD associated syndromes. Besides, we analyzed rare variants in a common disease-rare variant framework and performed rare variant burden analysis and gene enrichment analysis in phenotype based clusters.

**Results:**

We have diagnosed 13 molecularly proven syndromic autism cases. Strongest indicators of syndromic autism were intellectual disability, epilepsy or other neurological plus symptoms. Rare variant analysis on a cohort level confirmed the association of five genes with autism (*AUTS2*, *NHS*, *NSD1*, *SLC9A9*, and *VPS13*). We found no correlation between rare variant burden and number of minor malformation or autism severity. We identified four phenotypic clusters, but no specific gene was enriched in a given cluster.

**Conclusion:**

Our study indicates that NGS panel gene sequencing can be useful, where the clinical picture suggests a clinically defined syndromic autism. In this group, targeted panel sequencing may provide reasonable diagnostic yield. Unselected NGS panel screening in the clinic remains controversial, because of uncertain utility, and difficulties of the variant interpretation. However, the detected rare variants may still significantly influence autism risk and subphenotypes in a polygenic model, but to detect the effects of these variants larger cohorts are needed.

## Introduction

Autism spectrum disorder (ASD) is a neurodevelopmental disorder, characterized by the core symptoms of impaired social communication, restricted interests and stereotyped, repetitive behavior (Bourgeron, 2015). ASD has an estimated heritability of 64–91% (Woodbury-Smith and Scherer, 2018), suggesting a strong genetic effect, but the genetic background is highly heterogeneous (Vorstman et al., 2017). Common risk variants and rare variants both play a role (Bourgeron, 2015), and mutation types range from single nucleotide variants to large chromosomal aberrations, as well as variations in regulatory DNA elements (Turner et al., 2016). The number of genes implicated in ASD pathogenesis is >1,000 according to the SFARI database^1^, which converge on different cellular pathways (Krishnan et al., 2016). A shared genetic environment is possible with other psychiatric disorders as well, especially with schizophrenia (Brainstorm Consortium et al., 2018).

The clinical expression of ASD is nevertheless varied, despite the common umbrella term (Chaste et al., 2015). This high phenotypic variability, however, is mirrored poorly in the everyday clinical diagnostics of ASD. Indeed, DSM-5 now contains a single autism spectrum (Lord et al., 2018). This might be beneficial in the clinic but doesn’t serve the analysis of the genetic-phenotypic background. Earlier attempts to identify ASD subtypes or endophenotypes based on clinical features have met with limited success, and there were attempts to define subtypes based on genetics, which led for example to the identification of ASD with *CHD8* mutation subtype (Bernier et al., 2014). According to the classical definition, the syndromic ASD is a “disorder with a clinically defined pattern of somatic abnormalities and a neurobehavioral phenotype that may include ASD,” however, this is only present in 4–5% of cases (Fernandez and Scherer, 2017). Instead, if we move to a molecularly defined approach, in 25% of the cases there is a detectable strong genetic change, which may be even higher if multiple MPA are present (Fernandez and Scherer, 2017).

## Materials and Methods

In this study, we aimed to comprehensively analyze rare single nucleotides, and small INDEL variants in candidate ASD genes in a Hungarian ASD cohort, with NGS. Specifically, we tried to answer four questions in two hypothesis frameworks. On a case-by-case level: (1) How many patients can be detected with a probably strong acting variant (syndromic cases or Monogenic disorder) by targeted NGS gene sequencing? On a cohort level: (2) Is the detected number of variants in the tested genes significant? Does this confirm the pathogenicity status of the given genes? (3) Does rare variant burden associate with autism severity? (4) Do rare variants associate with any autism subphenotype?

### Patients

Autism spectrum disorder patients were recruited from the Vadaskert Child and Adolescent Psychiatry Hospital and Outpatient Clinic. Detailed clinical examinations consisting of a general medical examination and neurological assessment (NÁV, PB) were performed. A diagnosis of ASD was made by a qualified psychologist (CSP) using the ADI-R (Autism Diagnostic Interview-Revised) and ADOS (Autism Diagnostic Observation Schedule). Patients were screened for MPA, which were selected based on the Méhes Scale (Méhes, 1986). Family history and detailed environmental/societal data were collected from the parent (or parents) of each patient. Any disorders present in the parents, as well as environmental factors, were registered. The diagnosis of ASD was based on the standardized ADI-R in Hungarian, which was published by the Autism Foundation (Kapocs Publisher), according to the following scores: *A* ≥ 10 (social interaction), *B* ≥ 7 (communication), *C* ≥ 3 (repetitive stereotype manner), *D* ≥ 1 (abnormal development under 36 months).

### Genetic Analysis

DNA was isolated from peripheral blood samples from all participants using the QIAamp DNA blood kit (Qiagen, Hilden, Germany) according to the manufacturer’s instructions. We performed Fragile X-screening at every patient using the Amplidex *FMR1* PCR kit (Asuragen, Inc., Austin, TX, United States). Since Fragile-X-syndrome is a well-known cause of syndromic autism, and a unique molecular pathomechanism is probable, patients having *FMR1* gene alteration were excluded from all subsequent genetic analysis and phenotypic cluster analysis. The 101 ASD-associated genes (Betancur, 2011) were investigated with NGS, which was performed on a MiSeq (Illumina, San Diego, CA, United States) using the TruSight Autism Rapid Capture Kit (Illumina, San Diego, CA, United States) and the SureSelect QXT Kit (Agilent Technologies, Santa Clara, CA, United States) according to the manufacturer’s instructions. In the autism panel, 24 samples were multiplexed in a single run using the MiSeq reagent kit v2 and 300 cycles (Illumina, San Diego, CA, United States). The mean read depth was 135× in the gene panel, 20× coverage was achieved in a minimum of 90% of target regions. Pathogenic and likely pathogenic mutations from NGS data were validated by Sanger sequencing. Parents were Sanger sequenced for specific variants in selected cases, where syndromic ASD was suspected based on the sequencing results of the index case, and the parent was available for genetic analysis.

### Bioinformatic and Statistical Analysis

Raw sequences were filtered with Picard tools (version-2.1.0)^2^ and quality filtered reads were aligned to the hg19 reference genome with BWA-mem (Li and Durbin, 2009) using default parameters. Variant calling was performed using GATK HaplotypeCaller (version 3.3-0) (McKenna et al., 2010) and VCF files were annotated with SnpEff (version 4.1) (Cingolani et al., 2012). We analyzed only those variants that were found in the canonical transcripts of the gene. Variant quality was assessed by GATK, and only variants, which were flagged as PASS (RD >10, Mapping quality >40, quality by depth >2) were analyzed.

To filter potentially causal single-gene Mendelian variations on a case-by-case level, we used the VariantAnalyzer software developed at the Budapest University of Technology and Economics. This software application annotates SNPs and short INDELs with several types of annotations, such as their predicted function on genes using SnpEff, observed allele frequencies in several genomic projects including the 1000 Genomes Project and the ExAC, conservation scores based on PhyloP, PhastCons, GERP, predicted function of non-synonymous SNPs using dbNSFP, and disease associations with HGMD and ClinVar. By creating filter cascades based on these annotations and other information (e.g., genotypes and variant quality annotations), the software can easily be used to filter the variants through a user-friendly graphical interface. First, we filtered for variants known to be disease-causing, using the HGMD (Cooper et al., 1998), and ClinVar database (Landrum et al., 2014). Second, we filtered for rare variants based on the MAF and frequency of the mutation in our NGS data repository of 200 WES. Since large-scale genomic data of the Hungarian population is not available, a mutation with a low MAF may also be population-specific. We labeled a variant as rare if it was present maximum in one homozygous or two heterozygous samples within our ASD cohort of 174 patients (equal to a MAF-cutoff of ∼5‰), and the MAF in Europeans from the 1000 Genomes and ExAC databases, as well as in our in-house exome database was less than 5%. It is important to note that a limitation of this method is that it may exclude identification of founder mutations and disease-associated polymorphisms. Finally, mutations were prioritized based on their predicted effects. Exonic frameshifts, stop mutations and canonical splice site variants were considered damaging, whereas the effects of missense mutations were predicted using multiple prediction tools: SIFT (Sim et al., 2012), Polyphen2 (Adzhubei et al., 2010), CADD (Kircher et al., 2014), Radial SVM (Dong et al., 2015). The variants were assessed as recommended by the ACMG guideline 2015 (Richards et al., 2015). When siblings were analyzed for Mendelian disease-causing variants, both of them had to be carrier of the variant in question to be considered.

For the analysis of rare variants in a multifactorial hypothesis framework on a cohort level, the following methods were used. (1) We tested, whether the total number of detected rare missense or loss of function (stop, canonical splice site, and frameshift) variants in a given gene is greater than expected, with the method described by Rao and Nelson (2018). We filtered rare variants, with a MAF cut-off of 5% in the 1000 Genome European dataset, and in our internal exome database of 200 patients. P-value was calculated with the associated software: SORVA^3^. (2) For the calculation of rare variant burden, genes were normalized according to genic intolerance to mutation (Petrovski et al., 2013). Specifically, we used the inverse RVIS percentile [1–(RVIS percentile÷100)] accessed from http://genic-intolerance.org/, to give a weight to every gene than summed the number of variants in a given patient [Σ(variants × weighted-gene-score)]. For example, if a given gene was in the 2% percentile with RVIS, that means, that the gene got a weight of 0,98. When performing variant burden analysis we allowed to analyze siblings separately, because different rare variants might contribute to their phenotype, according to our hypothesis. Linear regression was used then to test for correlation between rare variant burden and autism severity, and rare variant burden vs. minor malformation burden. Autism severity was assessed by the total ADOS score, in patients, at whom ADOS was available (*N* = 47), and also by calibrated severity score using the method described by Gotham et al. (2009). For comparison of rare variant burden in males versus females, and the number of minor malformations in syndromic versus non-syndromic cases two-tailed *T*-test was used. (3) For the analysis of rare variant association with potential autism subphenotypes first, we assessed, whether such subphenotypes can be created based solely on the clinical data. We have used our clinical questionnaire containing 149 questions about family history, concomitant diseases, drugs, physical examination (neurologic and screening of minor malformations), and psychological status for cluster analysis. For the phenotypic cluster analysis, given our sample size and the low expected number of clusters, we utilized two kernel-based methods, namely kernel PCA and spectral clustering. Kernel methods have the additional benefit of being non-linear, i.e., able to identify non-linear combinations of clinical variables as relevant features. This requires the definition of a kernel matrix, which can be thought of as a pairwise similarity matrix over the samples, for which we used the mutual information as similarity measure in conjunction with the binarized clinical variables. In accordance with earlier studies, we set the number of clusters in spectral clustering to four. We also investigated the variables characterizing each cluster via computing the relative frequency of the presence of each feature. We performed the kernel PCA with 3 dimensions to visualize the transformed samples and cluster assignments. A natural question to ask is whether we can find a correlation between the subphenotypes and the genetic background, hinting at causal roles of genes in a given phenotype. In our case, the former is given by the clusters resulting from the questionnaire and the latter is given by the detected rare variants aggregated on candidate genes. To assess the correlation between the subphenotypes and genetics, we investigated whether detected rare variants of a candidate gene occur more frequently in either of the resulting clusters using ANOVA and pairwise *T*-tests, in conjunction with Bonferroni correction for multiple hypothesis testing.

## Results

### Phenotypic Description of the Cohort

One hundred and seventy-four individual with ASD [45 females and 129 males, median age = 6 years, interquartile range (IQR) = 7] were included in our study, including 5 sib-pairs. Clinical-demographic features of the cohort are presented in Table 1.

**Table 1:** Clinical-demographic features of the cohort.

Sex	M/F = 129/45 = 2.86/1
Median age (IQR)	6 (7)/5 (7)
**Average ADI**	
Reciprocal social interaction	23 ± 5,2
Communication (verbal/non-verbal)	13,95 ± 4,2/18,48 ± 2,5
Repetitive behavior	6,66 ± 2,1
**ADOS**	21,2 ± 5,7
Comorbid diagnosis	
ADHD (%)	84/174 (48,27%)
Epilepsy (%)	20/174 (11,49%)
Intellectual disability (%)	70/174 (40,22%)
**Ethnicity**	
Reported Hungarians	170
Reported Romani	2
Other	2
Maternal age at delivery (95% CI)	30,7 ± 1,05
Paternal age at delivery (95% CI)	33,4 ± 1,29
**Parent’s education level**	
College or higher at both parents	34%
College or higher at one parents	27%
High school or lower at both parents	39%


*The table describes the characteristics of the cohort. Ethnicity is self-reported. M/F is male to female ratio.*

**FIGURE 1:**
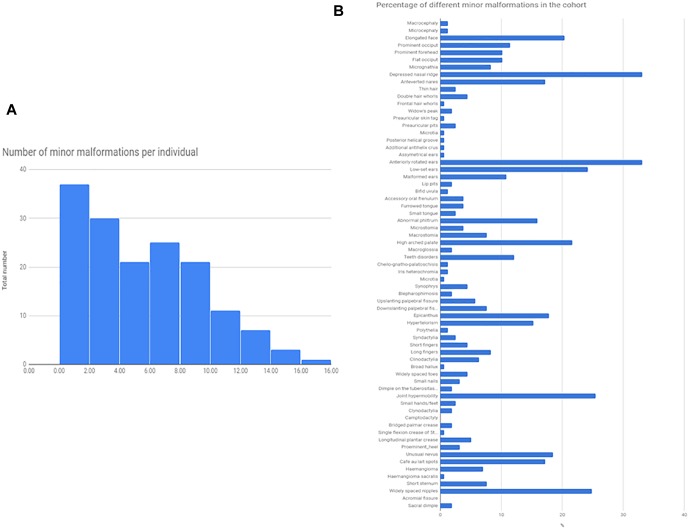
Total number of minor malformations, and the percentage of different minor malformations in the cohort. The Figure represents the total number of minor malformations/given individuals as a histogram **(A)**, and the prevalence of different minor malformations, as a percentage of the total cohort (*N* = 174) **(B)**.

Minor somatic abnormalities were frequent, at least one minor abnormality was present in 84% of the cohort from the Méhes scale. Histogram of the number of minor malformation per individual and the frequency of different minor malformations are shown in Figure 1. In general, molecularly diagnosed syndromic patients (13 cases, see next section) had on average no more minor malformations compared to non-syndromic cases (average number of minor malformations were 4.9/person in syndromic, and 5.03/person in non-syndromic cases, *T*-test *p* = 0.91). Strongest indicators of molecularly provable syndromic autism were ID, epilepsy or other neurological sign (such as ataxia), or a specific constellation of minor abnormalities as in the case of Fragile-X (FXS) and CHARGE syndrome.

**FIGURE 2:**
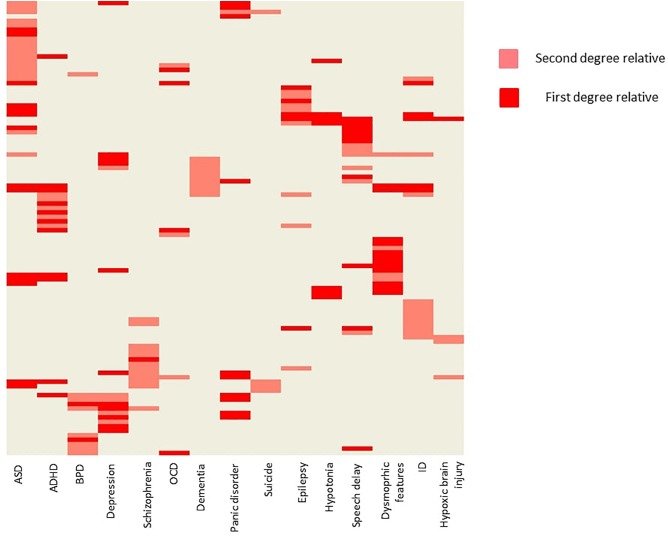
Family history of the probands. The figure is showing the probands with positive family history. Every row represents a single patient. Light red indicates, when a second-degree relative was affected with a given condition, and dark red indicates, when a first-degree relative was affected. Hierarchical clustering was applied on the heat map for better visualization. ADHD, attention deficit hyperactivity disorder; ASD, autism spectrum disorder; BPD, bipolar disorder; ID, intellectual disability; OCD, obsessive-compulsive disorder.

Positive family history (as reported by the parents) for psychiatric or neurological disorders were also common (Figure 2). ASD occurred at a first degree relative at 14 patients (8%), and at a second degree relative at 17 patients (9,7%). Besides psychiatric disorders, epilepsy (2,8% at first degree relatives, and 4,0% at second degree relatives), muscle hypotony (7 and 0%), speech development delay (10 and 6%), ID (5 and 11%), dysmorphic features (12 and 4%, respectively) were also common.

### Syndromic Forms of ASD

We have diagnosed 13 syndromic autism cases based on genetic findings (Table 2). Four patients were diagnosed with Fragile X syndrome, detected by screening, but these patients also had phenotypes consistent with FXS besides the definite autism. However, one of them is a girl (P1). In this case, the family history (2 healthy brothers), did not suggest an X-linked inheritance. Overall eight mutations were considered as pathogenic according to ACMG classification, with close phenotype match (four patients with FXS, one patient with Dravet syndrome, one patient with CHARGE syndrome, one patient with Duchenne muscular dystrophy, one patient with atypical Rett syndrome), and clear additional features (ID, epilepsy, muscular dystrophy, and multiple minor malformations) suggesting syndromic autism. The muscular dystrophy at the Duchenne patient (P7) was already known before the NGS study.

**Table 2:** Description of syndromic autism cases.

Patient	Sex	Phenotype_summary	Gene	Mutation	dbSNP	Zygosity	Inherited/*de novo*	Associated syndrome (inheritance pattern)	ACMG
P1	F	Mild ID, long face, low set ears, telecanthus, hypertelorism mammae, gothic palate, precocious puberty.	FMR1	CGG repeat full mutant	N/A	HET	*Unknown* (*parents not available for test*)	Fragile X syndrome (XL)	P
P2	M	Moderate ID, seizures, long face, low set large ears, prognathism, macroorchidism.	FMR1	CGG repeat full mutant	N/A	HEMI	Inherited (from mother, anticipation)	Fragile X syndrome (XL)	P
P3	M	Moderate ID, obsessive features, hand stereotypies, severely delayed speech, long face, low set, anteriorly rotated ears.	FMR1	CGG repeat full mutant	N/A	HEMI	Inherited (from mother, anticipation)	Fragile X syndrome (XL)	P
P4	M	Mild ID, frequent urinary tract infections, rectal prolapse, long face, low set anteriorly rotated ears, macroorchidism, micrognathia, impulsivity, mild tremor.	FMR1	CGG repeat mosaicism for premutation and full mutation	N/A	HEMI	*Unknown* (*parents not available for test*)	Fragile X syndrome (XL)	P
P5	M	Epileptic encephalopathy, ID, hypertelorism.	SCN1A	NM_001165963: c.4934G > A; p.R1645Q	rs121917976	HET	*De novo*	Dravet syndrome (AD)	P
P6	M	Mild hypotonia, failure to thrive, coloboma, low set ears, hypoplastic ears, atrial septum defect, severe ID, cryptorchidism, bifid nasal tips, micropenis, high lactate levels.	CHD7	NM_017780: c.4822delA; p.R1608Gfs^∗^31	.	HET	*De novo*	CHARGE syndrome (AD)	P
P7	M	Low set ears, gothic palate, epicanthus, muscular dystrophy.	DMD	NM_004006: c.8713C > T; p.R2905X	rs128627256	HEMI	Inherited (from mother)	Duchenne muscular dystrophy	P
P8	F	ID, prognathia, mild ataxia, hand stereotypes.	MECP2	NM_004992: c.763C > T; p.R255X	rs61749721	HET	*De novo*	Rett syndrome (XLD)	P
P9	F	Delayed psychomotor development, haemangioma, syndactyly, micrognathia, lower posterior hairline, anterior rotated, low set ears.	PTPN11	NM_002834: c.1502G > A; p.R501K	rs397507543	HET	*Unknown* (*mother is wild-type, father is not available for test*)	Noonan syndrome (AD)	LP
P10	F	Delayed language development, seizures, EEG: temporal lobe origin. Hypertelorism mammae, short neck, round face, large ear, synophrys, telecanthus, long philtrum, low posterior hairline.	RELN	NM_005045: c.2015C > T; p.P672L	rs201044262	HET	Inherited (from father)	Epilepsy, familial temporal lobe type 7 (AD)	LP
P11	F	Lissencephaly, hypertelorism, seizures, severe ID, prominent forehead, microcephaly, low set ears.	RELN	NM_005045: c.4354G > A; p.D1452N	rs114446781	HET	*De novo*	Lyssencephaly type 2 (AR)	LP
P12	F	Autoagression. No malformation, no spasticity. Mother has no spasticity.	SPAST	NM_014946: c.1625A > G; p.D542G	rs142053576	HET	Inherited (from mother)	Hereditary spastic paraparesis (AD)	VUS
P13	M	ID, ataxia, upsplanting palpebral fissure, small tongue, everted and thick upper lip, depressed nasal bridge, epilepsy.	AUTS2	NM_015570: c.2440GA > AT; p.E814M	.	HET	*Unknown* (*mother is wild-type, father is not available for test*)	Mental retardation 26 (AD)	VUS


*AD, autosomal dominant; AR, autosomal recessive; F, female; HEMI, hemizygous; HET, heterozygous; ID, intellectual disability; LP, likely pathogenic; M, male; P, pathogenic; VUS, variant of uncertain significance; XL, X-linked.*

One likely pathogenic mutation occurred in the *PTPN11* gene. The phenotype in this case (P9) was not typical for Noonan syndrome, however, the detected variant was reported earlier as pathogenic in ClinVar (RCV000037618.3) by a single submitter, the variant is rare (absent from 1000G and ExAc database, single occurrence in the cohort, absent from 200 Hungarian WES), predicted consequently as damaging by multiple prediction algorithms (SIFT, Polyphen, CADD, and MetaSVM), the gene is associated with dominant inheritance, and the variant is located in an evolutionarily conserved region (GERP score 5.13). Segregation analysis was not possible in this family. We detected two likely pathogenic variants in the *RELN* gene with different phenotypes. A heterozygous *RELN* variant (NM_005045: p.P672L) was present in a patient (P10) with epilepsy and multiple minor anomalies. This variant is also reported as pathogenic in the ClinVar database (RCV000193679.1). Segregation analysis proved that the variant is inherited from the father, who had no epilepsy, however, incomplete penetrance is possible according to the literature (OMIM:616436). At the case of P11, a *de novo* variant occurred in the *RELN* gene. The lissencephaly phenotype is associated with autosomal recessive inheritance according to the databases, but we classified this variant as likely pathogenic instead of a VUS, because of its proven *de novo* status. To rule out other possible genetic causes, an additional commercial lissencephaly panel testing was also performed (at Centogene, Rostock, Germany) which gave the same result.

**FIGURE 3:**
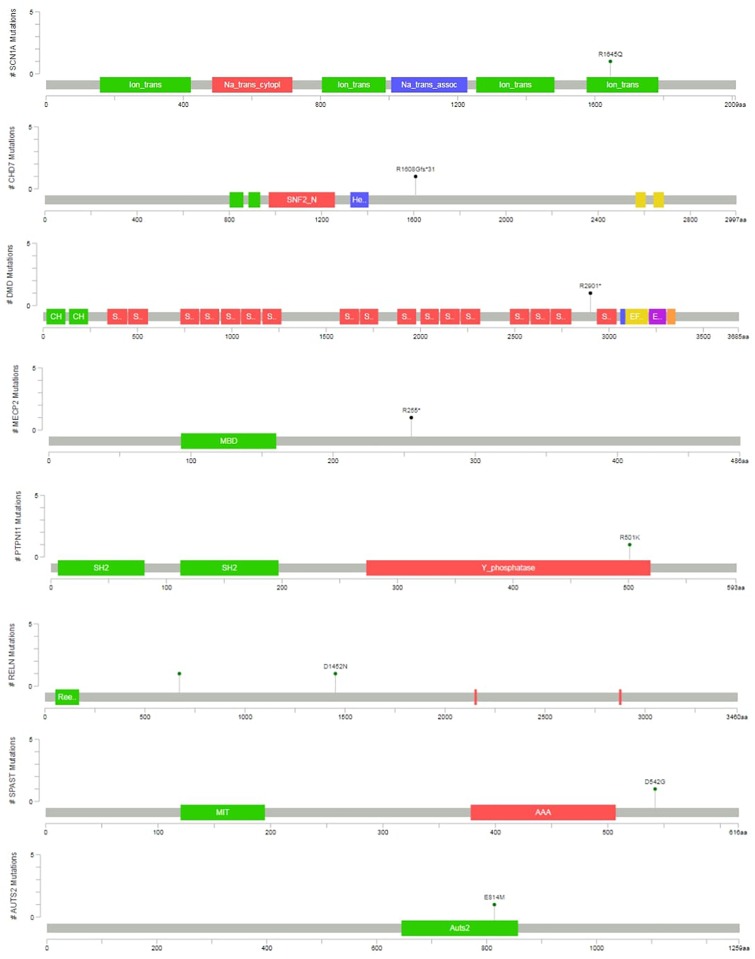
Variant position in syndromic cases. The graphical representation of the variants detected in syndromic ASD cases are presented. This was created with the mutation mapper software.

Two variants were classified as VUS. At the case of P12, the detected *SPAST* variant is reported as likely pathogenic in the ClinVar (RCV000199081.1), however, neither the patient nor the mother, who is also a carrier of the variant, have spasticity. In the case of P13, an unusual molecular event occurred. Two single nucleotide variants affected a single codon in the *AUTS2* (*KIAA0442*) gene (as proved by visualizing BAM files, and Sanger sequencing), resulting in p.E814M amino acid change. The phenotype is consistent with the literature (OMIM:615834), however, segregation analysis was not possible in the family. This patient died later as a consequence of a severe epileptic seizure. In total four variants was proved as *de novo*, and five variant was inherited. At four cases segregation analysis could not be performed in order to determine, whether the variant was *de novo* or inherited. The graphical representation of detected mutations in syndromic cases are presented^4^ in Figure 3.

### Cohort Level Number of Rare Variants in the Candidate Gene Panels

**FIGURE 4:**
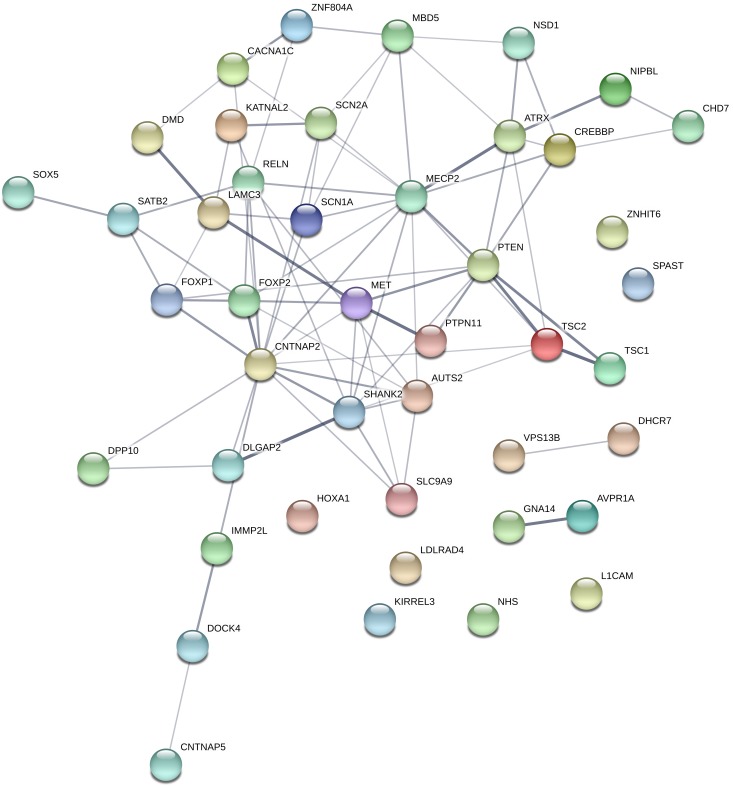
Genes, which contained rare, predicted pathogenic variants. Gene network representation of the genes, which harbored rare, predicted variants was created with STRING (https://string-db.org/). Edges represent interactions between the genes. It can be seen, that the interaction network contains a set of genes strongly connected (namely involved in nervous system development pathway GO:0007399; *p* = 3.22e-15), however, this is only suitable for demonstration purposes since the panel selection is a bias in the pathway analysis.

The total number of different rare variants (as defined in the section “Materials and Methods”), was 370. Among the 101 candidate gene, 80 genes contained rare variants, and 44 genes contained a rare predicted pathogenic variant (CADD score ≥ 20 OR SVM = damaging). These 44 genes are represented in Figure 4. Loss of function mutations occurred in 8 genes: *AUTS2*, *CHD7*, *DHCR7*, *DMD*, *GNA14*, *MECP2*, *SHANK2*, and *SHANK3* gene. Among these genes, *AUTS2*, *CHD7*, *SHANK2*, and *SHANK3* can be considered highly intolerant to functional variants (RVIS < 5 percentile), which suggest a pathogenic role for these genes. A table describing the number of different types of variants (missense, nonsense, frameshift INDEL, and non-frameshift INDEL) in all the investigated genes is provided as Supplementary Table S1.

We have used the software SORVA, with the method described by Rao and Nelson (2018) to assess whether the total number of rare missense and loss of function variants is greater than expected in the tested genes. Significantly greater than expected number of rare variants were detected in 5 of the 101 tested autism-linked genes: *AUTS2*, *NHS*, *NSD1*, *SLC9A9*, and *VPS13B* (Table 3). Rare variant burden did not differ between females and males (mean = 3.49 for males and 3.31 for females; *p* = 0,57). There was 67 individual (38.5%) in the cohort who did not carry any rare, predicted damaging variant in the candidate genes.

**Table 3:** Genes with an unexpectedly high number of rare variants.

Genes	Length_of_protein (AA)	Count_variants (variant/indiv + shared_variant/sib_pairs)	SORVA_p_value
*AUTS2*	1259	30/164 + 0/5	8.50E-06
*NHS*	1651	17/164 + 0/5	0.000149848233
*NSD1*	2696	53/164 + 0/5	8.40E-12
*SLC9A9*	645	17/164 + 1/5	0.026
*VPS13B*	4022	55/164 + 0/5	5.50E-05


*The table shows the genes, in which a significantly greater number than expected rare variant occurred. P-value was calculated with the SORVA software (MAF cut-off 5%). We summed the variants (heterozygous or homozygous) in the given gene in unrelated individuals (variant/indiv). In sib pairs, those variants were counted, which were shared (shared_variant/sib pairs).*

### Correlation of Rare Variant Burden With Autism Severity

**FIGURE 5:**
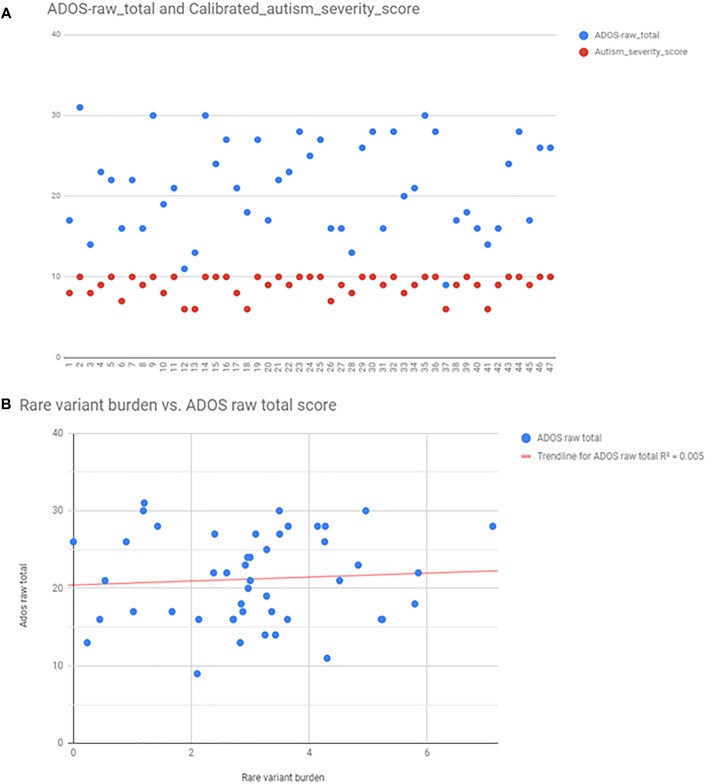
Autism severity scores calculated from ADOS, and the correlation between rare variant burden vs. ADOS scores. Panel **(A)** shows the scatter plot of ADOS raw total scores (blue points), and the calibrated severity scores (red points). Calculated scores were distributed only between 7 and 10, thus raw total scores were used for linear regression analysis **(B)**.

Autism severity was calculated by two methods: total raw ADOS score (available from 47 patients), and calculating calibrated severity score from ADOS raw total as described in the Section “Materials and Methods.” For the purpose of correlation analysis, we found that the calibrated severity score was not suitable, because most of the patients, who had ADOS fall into the most severe categories (8–10 score). ADOS raw total scores were better distributed among individuals (Figure 5), however, there was no correlation between ADOS raw total scores and rare variant burden by linear regression analysis (*R*^2^ = 0,0047; *p* = 0,648) (Figure 5). There was no correlation between rare variant burden and the number of minor malformations neither (*R*^2^ = 0,003; *p* = 0,51).

### Cluster Analysis and Gene Enrichment in the Phenotypic Clusters

**FIGURE 6:**
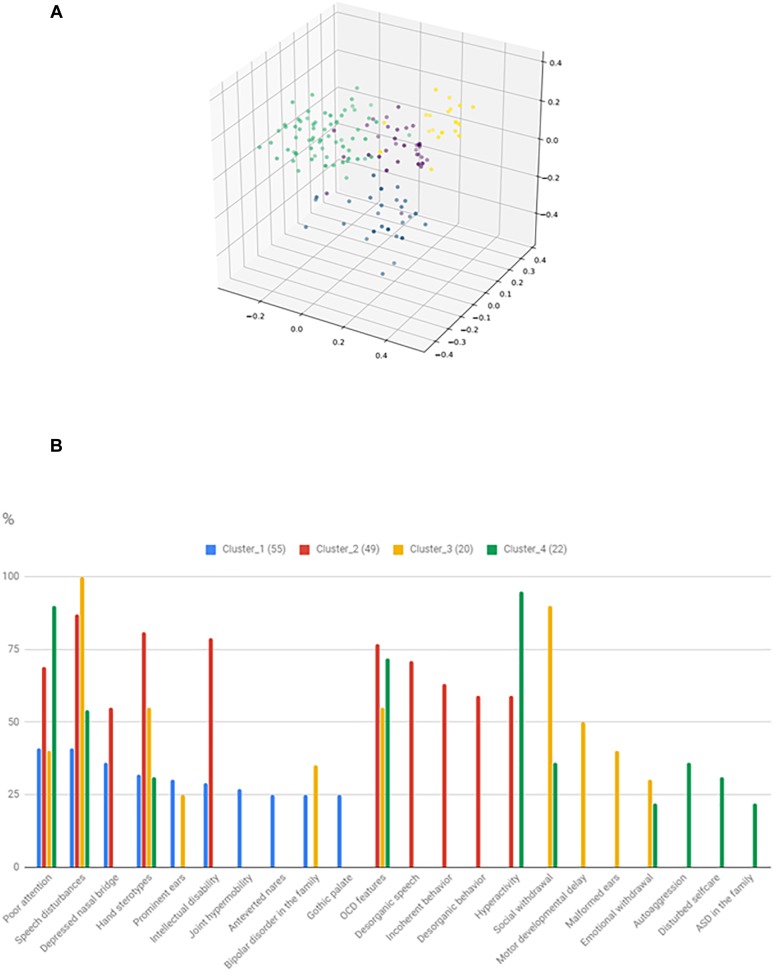
Result of the phenotypic cluster analysis. The three-dimensional figure **(A)** shows the result of kernel PCA with the identified phenotypic clusters. The histogram **(B)** represents the relative frequency of the 10 most common features in the given clusters.

The identified clusters and the most frequent 10 characterizing variables are shown in Figure 6. It can be seen that total 22 feature is involved in the 10 most common features of the clusters, thus significant overlap exists between the clusters. However, there are features, that particularly characterize certain clusters. Cluster_3 might be characterized by severe social disturbances, with prominent speech disturbance and social isolation. Cluster_4 is probably fitting into the ASD with comorbid ADHD category. Cluster_2 is characterized by ID plus frequent obsessive-compulsive (OCD) features with uncompensated behavior. Cluster_1 is the hardest to interpret, but one outstanding feature is the presence of BPD in the family and more than one minor physical anomaly. However, gene enrichment analysis did not show a significant overrepresentation of single genes in certain clusters. Syndromic cases were not enriched into a single cluster.

## Discussion

According to the literature, the overall heritability of ASD is estimated to be around 64–91% (Tick et al., 2016), suggesting a strong genetic factor in the background, but the underlying genetics seems to be very complex. The current models of autism genetics implicate a miscellaneous genetic environment, where common and rare variants act additively to create a risk for ASD (Weiner et al., 2017). According to Bourgeron’s hypothesis, common low-risk alleles set the background risk for ASD, and buffer the effects of rare deleterious variants (Bourgeron, 2015).

While it is estimated that common variants account for 40–60% of the overall liability of ASD (Klei et al., 2012), most genome-wide association (GWAS) studies were until now underpowered, and very few loci have been identified with genome-wide significance (Ramaswami and Geschwind, 2018). In contrast, hundreds of genes were described with rare variants, which significantly increase the risk of ASD, and it is estimated that they account for approximately 10–30% of autism cases (Fernandez and Scherer, 2017). Examples of evidence are an increased ASD risk with single rare variants of NLGN1 (Nakanishi et al., 2017), *VPS13B* (Ionita-Laza et al., 2014), *GABRB3* (Delahanty et al., 2011), and *PTCHD1* (Torrico et al., 2015). However, the contribution of these variants on a cohort level might be small. For example, a large WES study identified the strong risk factor gene *CHD8*, but pathogenic variants were only present in 15 out of 3,730 ASD cases (0.4%) (Bernier et al., 2014). For this reason, WES studies need very large sample sizes to reach a genome-wide significance (Liu et al., 2013). A possible solution to increase detection rate in WES studies is focusing on genes within shared haplotypes among individuals with ASD (Matsunami et al., 2014) or within regions that have been implicated by common variant GWAS analysis in large case-control cohorts, and collapsing individually rare variants on genes (burden-analysis) (Griswold et al., 2015).

In this study we utilized the method and software, developed by Rao and Nelson (2018), to test the gene level burden on a cohort level. This approach allowed us to use a general control group from earlier exome sequencing projects. In order to avoid population-specific variants, we excluded variants over 5% MAF in our in-house exome database. Significantly greater than expected number of rare variants were detected in five genes (*AUTS2*, *NHS*, *NSD1*, *SLC9A9*, and *VPS13B*), which confirm their role as a strong risk modifier gene. Due to our low cohort size, however, we could not confirm the roles of other candidates, or well-known ASD risk genes. We also tested, whether rare variant burden is correlated with autism severity, or the number of minor somatic abnormalities, but found no correlation. However, this might also be due to our low sample size.

The change of the view on the *AUTS2* gene is highlighting an important issue with rare variant interpretations in a complex disorder, like ASD. As knowledge was gathered, the view of *AUTS2* gene changed from an “autism susceptibility candidate gene” to a cause of a syndromic form of ASD (“AUTS2 syndrome”), with frequently associated ID, microcephaly and craniofacial abnormalities (Hori and Hoshino, 2017). We speculate, that this might also happen with other genes too. In our study, we identified loss of function mutations in the *GNA14*, *SHANK2*, and *SHANK3* genes, which are yet not linked to a characterized syndrome, but they are evolutionary intolerant to functional mutations. This makes genetic counseling very difficult in these cases. Further problems arise by the fact that ACMG interpretation is not suitable for the assessment of these genes (Richards et al., 2015), so there is no standardized framework for pathogenicity interpretation.

Many of the rare variants, detected in ASD studies, are *de novo*, according to the literature (O’Roak et al., 2011), and might act together with common polygenic risk variants, in a way, where rare variants modulate the ASD phenotype. Weiner et al. (2017) study showed that ASD cases with a strong acting *de novo* variant also carry a greater than expected polygenic risk, independently from the presence of the *de novo* variant. However, the presence of a *de novo* variant associated with an increased risk of co-occurring adverse neurodevelopmental outcomes (delayed walking, seizure, and ID). In our study, four of the 13 variants, linked to a syndromic autism case, were proved to be *de novo*. However, we could not perform segregation analyses in the whole cohort, for non-syndromic cases, to check whether the detected rare variants are *de novo* or not, therefore cannot draw further conclusions.

Due to the known challenges of phenotypic heterogeneity and variability of clinical presentation, identifying phenotypic subgroups has been a long-standing goal in ASD research (Prior et al., 1998). Previous studies indicate that objective approaches, such as dimensionality reduction methods (Tadevosyan-Leyfer et al., 2003) and cluster analysis (Verté et al., 2006; Hu and Steinberg, 2009; Hu and Lai, 2013) can be utilized in establishing homogeneous subgroups based on clinical features. In particular, linear methods, such as PCA, *k*-means and hierarchical clustering have been applied. Studies showed that the resulting clusters indeed characterized by certain symptoms (such as severe language deficits) and correlate with gene expression profiles (Hu and Steinberg, 2009). In this study, we identified four reasonable phenotypic clusters by spectral method, but could not find genes, which are enriched particularly in a given cluster. Syndromic cases were also not enriched into a single cluster.

The main purpose of phenotypic cluster analysis would be to potentially identify homogenous subgroups (endophenotypes), which may be linked to specific genetic background, pathogenesis, perhaps therapeutic approach. The phenotypic subgroups identified in this study show partial overlap with former studies. One of the strongest characterizing feature, which seems to be recurring in more studies is the presence of language impairment. Indeed it is also specified in the DSM-V (ASD with accompanying language disorder). Further distinctive features, which are clearly identifiable are association of ID, attention deficit and hyperactivity, and obsessive-compulsive features. It worth to mention, that many earlier attempts to identify ASD subgroups were mainly based on standard diagnostic questionnaires (Tadevosyan-Leyfer et al., 2003; Hu and Steinberg, 2009; Kienle et al., 2015; Cholemkery et al., 2016). The strength of this approach, that it utilizes existing resources to demonstrate, that subgroups may also be delineate on a spectrum disorder. However, there is a question whether this can provide new information. There is an opportunity also to use other resources too, such as electronic health records (Doshi-Velez et al., 2014), or brain imaging data (Hrdlicka et al., 2005). In our approach we used parental interview, and data on physical examination. On one hand we also have seen that language impairment, attention deficit, hyperactivity, and OCD features are important distinctive features. On the other hand we identified an additional cluster, where somatic malformation might be an outstanding feature. However, this need to be confirmed in larger samples, and possibly beneficial is a combined method (i.e., using standardized questionnaires together with additional data).

Besides the patients with rare genetic variants acting as a strong risk factor, a small group of ASD is called syndromic, where a single genetic variation (such as *FMR1* repeat expansion, *PTEN, TSC1*, or *MECP2* mutation) is considered as causal (Fernandez and Scherer, 2017). It is estimated that approximately 10–25% of individuals with ASD carry a highly penetrant genetic alteration, which might explain the phenotype (Bourgeron, 2015). In our cohort, we identified 13 syndromic patients (7,47% in total and 5,17% if we don’t consider Fragile X patients detected by screening). This is somewhat lower than the above-mentioned average, however, we only analyzed single nucleotide variants and small INDELs, which is a limitation of this study. In a recent study, microarray, Fragile X testing and targeted gene panel testing was performed in 100 children with ASD (Kalsner et al., 2018). Copy number variants believed to contribute to ASD risk were identified in 12%, but the gene panel did not increase the diagnostic yield. It has to be mentioned although, that rare variants, not considered as causing a syndrome, might also significantly modify the phenotype (such as IQ, head size) (Pizzo et al., 2018). This again draws the attention to the issue that the clinical interpretation of these variants may significantly differ among laboratories. It has to be mentioned also, that none of the identified syndromic cases was unexpected, in a sense that all of them had additional signs suggesting syndromic autism ab ovo.

Potential clinical translation of genetic diagnosis can be manifold. In the syndromic group it helps to estimate recurrence risk, the prognosis, potential specific comorbidities, and therapeutic approaches. Taking these into account, well-proved syndromic ASD-associated genes need to be investigated at least in the clinically defined ASD-group. The phenotypic clustering can potentially help to correctly identify which group has to be tested. In the group of non-syndromic ASD the indication for genetic investigation is currently less clear. In this group the simultaneous effect of the different common and rare variants should be estimated, which is currently dubious. Polygenic risk scores are emerging as a tool to predict disease risk in multifactorial diseases, but have many potential bias (De La Vega and Bustamante, 2018; Torkamani et al., 2018). Even so polygenic risk score show promise, for example earlier studies showed that ASD polygenic risk score is positively correlated with general cognitive ability (Clarke et al., 2016), or with specific fMRI connectivity pattern (Wang et al., 2017). However, we are far away from everyday clinical utilization.

Certain limitations of the study should be mentioned. The size of the cohort is perhaps the most important one. As we looked specifically for rare variants in a relatively small cohort, the probability of finding variants with very low MAF is low. This could explain our result, that only five gene had significantly more rare variant burden than expected by spontaneous mutation rate, gene size and genic intolerance. This can result in false negative findings. False positive findings may also arise due to small sample size, however, this is mitigated by the fact, that well established ASD-genes were included in the panel. However, this approach has also disadvantages. The low number of total variant detected made impossible to analyze ethnicity and cryptic relatedness from the genetic data. On the other hand selection of a gene panel always contains a bias, since the number of genes linked to ASD is around 1000 according to the SFARI database^5^. However, many of these do not have an associated Mendelian disorder. Finally we did not carry out functional experiments in this study, so cannot distinct clearly between rare non-functional and rare functional variants. We used protein prediction scores to assess the probability of functional impact of a variant.

## Conclusion

In this study, we performed an analysis of rare single nucleotide and small INDEL variants in a Hungarian ASD cohort, detected by NGS panel testing, in order to identify syndromic autism cases and to assess the contribution of rare variants in formerly established ASD genes on a cohort level. Our study indicates that NGS panel gene sequencing can be useful in dedicated cases, where the clinical picture suggests a clinically defined syndromic autism (i.e., associated ID, epilepsy, neurological signs, a certain pattern of somatic malformation, or positive family history). In this group, targeted panel sequencing may provide reasonable diagnostic yield. However, the necessity of unselected NGS panel screening in the clinic remains controversial, because of uncertain clinical utility, and difficulties of the variant interpretation. The detected rare variants may still significantly influence autism risk and subphenotypes in a polygenic model. However, to detect the effects of these variants large cohorts are needed. As knowledge will increase about the contribution of these rare variants on the phenotype, an individual assessment might also be beneficial in the future for personalized management of patients with ASD.

## Ethics Statement

Written informed consent was obtained from the parents of the patient, or over 18 years of age, directly from the patients. This study was performed in accordance with the Helsinki Declaration of 1975 and was approved by the Hungarian Research Ethics Committee (44599-2/2013/EKU).

## Author Contributions

MM, PB, and NV designed the study. NV and PB performed data collection and physical examination of the patients. KP performed the next generations sequencing. PB and NV analyzed the NGS data. AGé provided the bioinformatical platform to analyze the NGS data and provided bioinformatical support for the study. RB and AGá made the validation by Sanger sequencing and the segregation analysis of the family members. CP performed the neuropsychological testing of the patients. BB, PB, and VM performed the statistical analyses. PB, NV, and MM wrote the manuscript. MM coordinated the research team, led the manuscript preparation, and revised and corrected the manuscript. All authors read and approved the final manuscript.

## Conflict of Interest Statement

The authors declare that the research was conducted in the absence of any commercial or financial relationships that could be construed as a potential conflict of interest.

## Footnotes

1 https://gene.sfari.org/

2 http://broadinstitute.github.io/picard/

3 https://sorva.genome.ucla.edu/

4 http://mutationmapper.bioch.ox.ac.uk/

5 https://gene.sfari.org/

## Supplementary Material

Table S1 Number of variants in the investigated genes. Table summarizes the number of different variant types, detected in the investigated genes. Table only includes rare variants, as defined in the Section “Materials and Methods.” n/a, not applicable. Next generation sequencing is not suitable for detection of repeat expansion events. Repeat expansions were only investigated in the FMR1 gene, in order to diagnose Fragile-X patients.

## Abbreviations

ACMG: American College of Medical Genetics
ADHD: attention deficit hyperactivity disorder
ADI-R: Autism Diagnostic Interview – Revised
ADOS: Autism Diagnostic Observation Schedule
ASD: autism spectrum disorder
BPD: bipolar disorder
CHARGE: coloboma, heart defects, atresia choanae, growth retardation, genital abnormalities, and ear abnormalities
ExAC: Exome Aggregation Consortium
FXS: Fragile-X syndrome
GWAS: genome wide association study
HGMD: Human Gene Mutation Database
ID: intellectual disability
INDEL: insertion/deletion
MAF: minor allele frequency
MPA: minor physical anomalies
NGS: next generation sequencing
OCD: obsessive compulsive disorder
PCA: principal component analysis
VUS: variant with uncertain significance
WES: whole exome sequencing

## References

[B1] Adzhubei I. A. Schmidt S. Peshkin L. Ramensky V. E. Gerasimova A. Bork P. (2010). A method and server for predicting damaging missense mutations. *Nat. Methods* 7 248–249. 10.1038/nmeth0410-248 20354512 PMC2855889

[B2] Bernier R. Golzio C. Xiong B. Stessman H. A. Coe B. P. Penn O. (2014). Disruptive CHD8 mutations define a subtype of autism early in development. *Cell* 158 263–276. 10.1016/j.cell.2014.06.017 24998929 PMC4136921

[B3] Betancur C. (2011). Etiological heterogeneity in autism spectrum disorders: more than 100 genetic and genomic disorders and still counting. *Brain Res.* 1380 42–77. 10.1016/j.brainres.2010.11.078 21129364

[B4] Bourgeron T. (2015). From the genetic architecture to synaptic plasticity in autism spectrum disorder. *Nat. Rev. Neurosci.* 16 551–563. 10.1038/nrn3992 26289574

[B5] Brainstorm Consortium V. Anttila V. Bulik-Sullivan B. Finucane H. K. Walters R. K. Bras J. (2018). Analysis of shared heritability in common disorders of the brain. *Science* 360:eaa8757. 10.1126/science.aap8757 29930110 PMC6097237

[B6] Chaste P. Klei L. Sanders S. J. Hus V. Murtha M. T. Lowe J. K. (2015). A genome-wide association study of autism using the simons simplex collection: does reducing phenotypic heterogeneity in autism increase genetic homogeneity? *Biol. Psychiatry* 77 775–784. 10.1016/j.biopsych.2014.09.017 25534755 PMC4379124

[B7] Cholemkery H. Medda J. Lempp T. Freitag C. M. (2016). Classifying autism spectrum disorders by ADI-R: subtypes or severity gradient? *J. Autism Dev. Disord.* 46 2327–2339. 10.1007/s10803-016-2760-2 26956715

[B8] Cingolani P. Platts A. Wang L. L. Coon M. Nguyen T. Wang L. (2012). A program for annotating and predicting the effects of single nucleotide polymorphisms, SnpEff: SNPs in the genome of *Drosophila melanogaster* strain w 1118; iso-2; iso-3. *Fly* 6 80–92. 10.4161/fly.19695 22728672 PMC3679285

[B9] Clarke T. K. Lupton M. K. Fernandez-Pujals A. M. Starr J. Davies G. Cox S. (2016). Common polygenic risk for autism spectrum disorder (ASD) is associated with cognitive ability in the general population. *Mol. Psychiatry* 21 419–425. 10.1038/mp.2015.12 25754080 PMC4759203

[B10] Cooper D. N. Ball E. V. Krawczak M. (1998). The human gene mutation database. *Nucleic Acids Res.* 26 285–287. 10.1093/nar/26.1.285 9399854 PMC147254

[B11] De La Vega F. M. Bustamante C. D. (2018). Polygenic risk scores: a biased prediction? *Genome Med.* 10:100. 10.1186/s13073-018-0610-x PMC6309089 30591078

[B12] Delahanty R. J. Kang J. Q. Brune C. W. Kistner E. O. Courchesne E. Cox N. J. (2011). Maternal transmission of a rare GABRB3 signal peptide variant is associated with autism. *Mol. Psychiatry* 16 86–96. 10.1038/mp.2009.118 19935738 PMC3428055

[B13] Dong C. Wei P. Jian X. Gibbs R. Boerwinkle E. Wang K. (2015). Comparison and integration of deleteriousness prediction methods for nonsynonymous SNVs in whole exome sequencing studies. *Hum. Mol. Genet.* 24 2125–2137. 10.1093/hmg/ddu733 25552646 PMC4375422

[B14] Doshi-Velez F. Ge Y. Kohane I. (2014). Comorbidity clusters in autism spectrum disorders: an electronic health record time-series analysis. *Pediatrics* 133 e54–e63. 10.1542/peds.2013-0819 24323995 PMC3876178

[B15] Fernandez B. A. Scherer S. W. (2017). Syndromic autism spectrum disorders: moving from a clinically defined to a molecularly defined approach. *Dialogues Clin. Neurosci.* 19 353–371. 29398931 10.31887/DCNS.2017.19.4/sscherer PMC5789213

[B16] Gotham K. Pickles A. Lord C. (2009). Standardizing ADOS scores for a measure of severity in autism spectrum disorders. *J. Autism Dev. Disord.* 39 693–705. 10.1007/s10803-008-0674-3 19082876 PMC2922918

[B17] Griswold A. J. Dueker N. D. Van Booven D. Rantus J. A. Jaworski J. M. Slifer S. H. (2015). Targeted massively parallel sequencing of autism spectrum disorder-associated genes in a case control cohort reveals rare loss-of-function risk variants. *Mol. Autism* 6:43. 10.1186/s13229-015-0034-z 26185613 PMC4504419

[B18] Hori K. Hoshino M. (2017). Neuronal migration and AUTS2 syndrome. *Brain Sci.* 7:54. 10.3390/brainsci7050054 28505103 PMC5447936

[B19] Hrdlicka M. Dudova I. Beranova I. Lisy J. Belsan T. Neuwirth J. (2005). Subtypes of autism by cluster analysis based on structural MRI data. *Eur. Child Adolesc. Psychiatry* 14 138–144. 10.1007/s00787-005-0453-z 15959659

[B20] Hu V. W. Lai Y. (2013). Developing a predictive gene classifier for autism spectrum disorders based upon differential gene expression profiles of phenotypic subgroups. *N. Am. J. Med. Sci.* 6 107–116. 10.7156/najms.2013.0603107 24363828 PMC3867975

[B21] Hu V. W. Steinberg M. E. (2009). Novel clustering of items from the autism diagnostic interview-revised to define phenotypes within autism spectrum disorders. *Autism Res.* 2 67–77. 10.1002/aur.72 19455643 PMC2737479

[B22] Ionita-Laza I. Capanu M. De Rubeis S. McCallum K. Buxbaum J. D. (2014). Identification of rare causal variants in sequence-based studies: methods and applications to VPS13B, a gene involved in Cohen syndrome and autism. *PLoS Genet.* 10:e1004729. 10.1371/journal.pgen.1004729 25502226 PMC4263785

[B23] Kalsner L. Twachtman-Bassett J. Tokarski K. Stanley C. Dumont-Mathieu T. Cotney J. (2018). Genetic testing including targeted gene panel in a diverse clinical population of children with autism spectrum disorder: findings and implications. *Mol. Genet. Genomic Med.* 6 171–185. 10.1002/mgg3.354 29271092 PMC5902398

[B24] Kienle X. Freiberger V. Greulich H. Blank R. (2015). Autism spectrum disorder and DSM-5: spectrum or cluster? *Prax Kinderpsychol. Kinderpsychiatr.* 64 412–428. 10.13109/prkk.2015.64.6.412 26289149

[B25] Kircher M. Witten D. M. Jain P. O’Roak B. J. Cooper G. M. Shendure J. (2014). A general framework for estimating the relative pathogenicity of human genetic variants. *Nat. Genet.* 46 310–315. 10.1038/ng.2892 24487276 PMC3992975

[B26] Klei L. Sanders S. J. Murtha M. T. Hus V. Lowe J. K. Willsey A. J. (2012). Common genetic variants, acting additively, are a major source of risk for autism. *Mol. Autism* 3:9. 10.1186/2040-2392-3-9 23067556 PMC3579743

[B27] Krishnan A. Zhang R. Yao V. Theesfeld C. L. Wong A. K. Tadych A. (2016). Genome-wide prediction and functional characterization of the genetic basis of autism spectrum disorder. *Nat. Neurosci.* 19 1454–1462. 10.1038/nn.4353 27479844 PMC5803797

[B28] Landrum M. J. Lee J. M. Riley G. R. Jang W. Rubinstein W. S. Church D. M. (2014). ClinVar: public archive of relationships among sequence variation and human phenotype. *Nucleic Acids Res.* 42 D980–D985. 10.1093/nar/gkt1113 24234437 PMC3965032

[B29] Li H. Durbin R. (2009). Fast and accurate short read alignment with burrows-Wheeler transform. *Bioinformatics* 25 1754–1760. 10.1093/bioinformatics/btp324 19451168 PMC2705234

[B30] Liu L. Sabo A. Neale B. M. Nagaswamy U. Stevens C. Lim E. (2013). Analysis of rare, exonic variation amongst subjects with autism spectrum disorders and population controls. *PLoS Genet.* 9:e1003443. 10.1371/journal.pgen.1003443 23593035 PMC3623759

[B31] Lord C. Elsabbagh M. Baird G. Veenstra-Vanderweele J. (2018). Autism spectrum disorder. *Lancet* 392 508–520. 10.1016/S0140-6736(18)31129-2 30078460 PMC7398158

[B32] Matsunami N. Hensel C. H. Baird L. Stevens J. Otterud B. Leppert T. (2014). Identification of rare DNA sequence variants in high-risk autism families and their prevalence in a large case/control population. *Mol. Autism* 5:5. 10.1186/2040-2392-5-5 24467814 PMC4098669

[B33] McKenna A. Hanna M. Banks E. Sivachenko A. Cibulskis K. Kernytsky A. (2010). The genome analysis toolkit: a mapReduce framework for analyzing next-generation DNA sequencing data. *Genome Res.* 20 1297–1303. 10.1101/gr.107524.110 20644199 PMC2928508

[B34] Méhes K. (1986). [Informative morphogenetic variants (minor congenital anomalies)]. *Orv. Hetil.* 127 3001–3003. 3796998

[B35] Nakanishi M. Nomura J. Ji X. Tamada K. Arai T. Takahashi E. (2017). Functional significance of rare neuroligin 1 variants found in autism. *PLoS Genet.* 13:e1006940. 10.1371/journal.pgen.1006940 28841651 PMC5571902

[B36] O’Roak B. J. Deriziotis P. Lee C. Vives L. Schwartz J. J. Girirajan S. (2011). Exome sequencing in sporadic autism spectrum disorders identifies severe de novo mutations. *Nat. Genet.* 43 585–589. 10.1038/ng.835 21572417 PMC3115696

[B37] Petrovski S. Wang Q. Heinzen E. L. Allen A. S. Goldstein D. B. (2013). Genic intolerance to functional variation and the interpretation of personal genomes. *PLoS Genet.* 9:e1003709. 10.1371/journal.pgen.1003709 23990802 PMC3749936

[B38] Pizzo L. Jensen M. Polyak A. Rosenfeld J. A. Mannik K. Krishnan A. (2018). Rare variants in the genetic background modulate cognitive and developmental phenotypes in individuals carrying disease-associated variants. *Genet. Med.* 21 816–825. 10.1038/s41436-018-0266-3 30190612 PMC6405313

[B39] Prior M. Eisenmajer R. Leekam S. Wing L. Gould J. Ong B. (1998). Are there subgroups within the autistic spectrum? A cluster analysis of a group of children with autistic spectrum disorders. *J. Child Psychol. Psychiatry* 39 893–902. 10.1017/s0021963098002807 9758197

[B40] Ramaswami G. Geschwind D. H. (2018). Genetics of autism spectrum disorder. *Handb. Clin. Neurol.* 147 321–329. 10.1016/B978-0-444-63233-3.00021-X 29325621

[B41] Rao A. R. Nelson S. F. (2018). Calculating the statistical significance of rare variants causal for mendelian and complex disorders. *BMC Med. Genomics* 11:53. 10.1186/s12920-018-0371-9 29898714 PMC6001062

[B42] Richards S. Aziz N. Bale S. Bick D. Das S. Gastier-Foster J. (2015). Standards and guidelines for the interpretation of sequence variants: a joint consensus recommendation of the American college of medical genetics and genomics and the association for molecular pathology. *Genet. Med.* 17 405–423. 10.1038/gim.2015.30 25741868 PMC4544753

[B43] Sim N.-L. Kumar P. Hu J. Henikoff S. Schneider G. Ng P. C. (2012). SIFT web server: predicting effects of amino acid substitutions on proteins. *Nucleic Acids Res.* 40 W452–W457. 10.1093/nar/gks539 22689647 PMC3394338

[B44] Tadevosyan-Leyfer O. Dowd M. Mankoski R. Winklosky B. Putnam S. McGrath L. (2003). A principal components analysis of the autism diagnostic interview-revised. *J. Am. Acad. Child Adolesc. Psychiatry* 42 864–872. 12819447 10.1097/01.CHI.0000046870.56865.90

[B45] Tick B. Bolton P. Happé F. Rutter M. Rijsdijk F. (2016). Heritability of autism spectrum disorders: a meta-analysis of twin studies. *J. Child Psychol. Psychiatry* 57 585–595. 10.1111/jcpp.12499 26709141 PMC4996332

[B46] Torkamani A. Wineinger N. E. Topol E. J. (2018). The personal and clinical utility of polygenic risk scores. *Nat. Rev. Genet.* 19 581–590. 10.1038/s41576-018-0018-x 29789686

[B47] Torrico B. Fernàndez-Castillo N. Hervás A. Milà M. Salgado M. Rueda I. (2015). Contribution of common and rare variants of the PTCHD1 gene to autism spectrum disorders and intellectual disability. *Eur. J. Hum. Genet.* 23 1694–1701. 10.1038/ejhg.2015.37 25782667 PMC4795195

[B48] Turner T. N. Hormozdiari F. Duyzend M. H. McClymont S. A. Hook P. W. Iossifov I. (2016). Genome sequencing of autism-affected families reveals disruption of putative noncoding regulatory DNA. *Am. J. Hum. Genet.* 98 58–74. 10.1016/j.ajhg.2015.11.023 26749308 PMC4716689

[B49] Verté S. Geurts H. M. Roeyers H. Rosseel Y. Oosterlaan J. Sergeant J. A. (2006). Can the children’s communication checklist differentiate autism spectrum subtypes? *Autism* 10 266–287. 10.1177/1362361306063299 16682398

[B50] Vorstman J. A. S. Parr J. R. Moreno-De-Luca D. Anney R. J. L. Nurnberger J. I. Hallmayer J. F. (2017). Autism genetics: opportunities and challenges for clinical translation. *Nat. Rev. Genet. Mar.* 6 362–376. 10.1038/nrg.2017.4 28260791

[B51] Wang T. Zhang X. Li A. Zhu M. Liu S. Qin W. (2017). Polygenic risk for five psychiatric disorders and cross-disorder and disorder-specific neural connectivity in two independent populations. *Neuroimage Clin.* 14 441–449. 10.1016/j.nicl.2017.02.011 28275544 PMC5328751

[B52] Weiner D. J. Wigdor E. M. Ripke S. Walters R. K. Kosmicki J. A. Grove J. (2017). Polygenic transmission disequilibrium confirms that common and rare variation act additively to create risk for autism spectrum disorders. *Nat. Genet.* 49 978–985. 10.1038/ng.3863 28504703 PMC5552240

[B53] Woodbury-Smith M. Scherer S. W. (2018). Progress in the genetics of autism spectrum disorder. *Dev. Med. Child Neurol.* 60 445–451. 10.1111/dmcn.13717 29574884

