# Supplementary material for: Comprehensive Analysis of Rare Variants of 101 Autism-Linked Genes in a Hungarian Cohort of Autism Spectrum Disorder Patients
Source: Front Genet. 2019 May 8;10:434. doi: 10.3389/fgene.2019.00434 (PMC6517558; doi:10.3389/fgene.2019.00434)
Supplement: Table S1 — Number of variants in the investigated genes. Table summarizes the number of different variant types, detected in the investigated genes. Table only includes rare variants, as defined in the Section “Materials and Methods.” n/a, not applicable. Next generation sequencing is not suitable for detection of repeat expansion events. Repeat expansions were only investigated in the FMR1 gene, in order to diagnose Fragile-X patients. [file Table_1.docx]

|  | *Missense variant* | *Missense variant with damaging prediction scores* | *Nonsense variant* | *Frameshift INDEL* | *Non-frameshift (Codon) INDEL* | *Repeat expansion* |
| --- | --- | --- | --- | --- | --- | --- |
| ANKRD11 | **16** | - | - | - | **1** | n/a |
| AP1S2 | - | - | - | - | - | n/a |
| ARX | - | - | - | - | - | n/a |
| ATRX | **3** | **1** | - | - | - | n/a |
| AUTS2 | **8** | **4** | - | **1** | - | n/a |
| AVPR1A | **1** | **1** | - | - | - | n/a |
| BDNF | **1** | - | - | - | - | n/a |
| BRAF | **1** | - | - | - | - | n/a |
| LDLRAD4 | **3** | **2** | - | - | - | n/a |
| CACNA1C | **6** | **4** | - | - | - | n/a |
| CASK | - | - | - | - | - | n/a |
| CDKL5 | **1** | - | - | - | - | n/a |
| CHD7 | **5** | **1** | - | **1** | **2** | n/a |
| CHD8 | **5** | - | - | - | **1** | n/a |
| CNTNAP2 | **4** | **1** | - | - | - | n/a |
| CNTNAP5 | **9** | **1** | - | - | - | n/a |
| CREBBP | **9** | **1** | - | - | - | n/a |
| DHCR7 | **6** | **6** | **1** | - | - | n/a |
| DLGAP2 | **6** | **1** | - | - | - | n/a |
| DMD | **17** | **2** | **1** | **1** | **1** | n/a |
| DOCK4 | **8** | **1** | - | - | - | n/a |
| DPP10 | **4** | **1** | - | - | - | n/a |
| DPP6 | **3** | - | - | - | - | n/a |
| EHMT1 | **6** | - | - | - | - | n/a |
| FGD | - | - | - | - | - | n/a |
| FMR1 | **1** | - | - | - | - | **4** |
| FOLR1 | **3** | - | - | - | - | n/a |
| FOXG1 | - | - | - | - | - | n/a |
| FOXP1 | **1** | **1** | - | - | - | n/a |
| FOXP2 | **3** | **1** | - | - | **1** | n/a |
| GABRB3 | - | - | - | - | - | n/a |
| GABRG1 | **2** | - | - | - | - | n/a |
| GNA14 | **6** | **4** | - | **1** | - | n/a |
| GRIN2B | - | - | - | - | - | n/a |
| GRPR | **3** | - | - | - | - | n/a |
| HOXA1 | **3** | **1** | - | - | - | n/a |
| HPRT1 | - | - | - | - | - | n/a |
| IMMP2L | **2** | **1** | - | - | - | n/a |
| KATNAL2 | **2** | **1** | - | - | - | n/a |
| KCTD13 | - | - | - | - | - | n/a |
| KDM5C | **2** | - | - | - | - | n/a |
| KIRREL3 | **1** | **1** | - | - | - | n/a |
| KLHL3 | - | - | - | - | - | n/a |
| L1CAM | **1** | - | - | - | - | n/a |
| LAMC3 | **12** | **2** | - | - | - | n/a |
| MBD5 | **3** | **1** | - | - | - | n/a |
| MECP2 | **3** | **1** | **1** | - | 1 | n/a |
| MED12 | **1** | - | - | - | - | n/a |
| MEF2C | - | - | - | - | - | n/a |
| MET | **7** | **1** | - | - | - | n/a |
| MID1 | - | - | - | - | - | n/a |
| NEGR1 | **3** | - | - | - | - | n/a |
| NHS | **5** | **1** | - | - | - | n/a |
| NIPBL | **3** | **2** | - | - | - | n/a |
| NLGN3 | **2** | - | - | - | - | n/a |
| NLGN4X | **1** | - | - | - | - | n/a |
| NRXN1 | **4** | - | - | - | - | n/a |
| NSD1 | **12** | **2** | - | - | - | n/a |
| NTNG1 | **1** | - | - | - | - | n/a |
| OPHN1 | - | - | - | - | - | n/a |
| PAFAH1B1 | - | - | - | - | - | n/a |
| PCDH19 | **4** | - | - | - | - | n/a |
| PCDH9 | **3** | - | - | - | - | n/a |
| PDE10A | **3** | - | - | - | - | n/a |
| PHF6 | - | - | - | - | - | n/a |
| PIP5K1B | **2** | - | - | - | - | n/a |
| PNKP | **2** | - | - | - | - | n/a |
| PON3 | **4** | **1** | - | - | - | n/a |
| PQBP1 | - | - | - | - | - | n/a |
| PTCHD1 | **1** | - | - | - | - | n/a |
| PTEN | **1** | **1** | - | - | - | n/a |
| PTPN11 | **1** | **1** | - | - | - | n/a |
| RAB39B | - | - | - | - | - | n/a |
| RAI1 | **8** | - | - | - | **1** | n/a |
| RBFOX1 | **2** | - | - | - | - | n/a |
| RELN | **18** | **12** | - | - | - | n/a |
| RPL10 | - | - | - | - | - | n/a |
| SATB2 | **3** | **2** | - | - | - | n/a |
| SCN1A | **2** | **1** | - | - | - | n/a |
| SCN2A | **5** | **4** | - | - | - | n/a |
| SHANK2 | **15** | **5** | **1** | - | **1** | n/a |
| SHANK3 | **5** | - | - | **2** | - | n/a |
| SLC6A4 | **2** | - | - | - | - | n/a |
| SLC9A6 | **1** | - | - | - | - | n/a |
| SLC9A9 | **5** | **2** | - | - | - | n/a |
| SMC1A | - | - | - | - | - | n/a |
| SMG6 | **2** | - | - | - | - | n/a |
| SNRPN | - | - | - | - | - | n/a |
| SOX5 | **1** | **1** | - | - | - | n/a |
| SPAST | **5** | **2** | - | - | - | n/a |
| ST7 | **2** | - | - | - | - | n/a |
| STK3 | **1** | **1** | - | - | - | n/a |
| TCF4 | - | - | - | - | - | n/a |
| TSC1 | **3** | **1** | - | - | - | n/a |
| TSC2 | **1-** | **2** | - | - | **1** | n/a |
| UBE3A | **1** | - | - | - | - | n/a |
| VPS13B | **24** | **5** | - | - | - | n/a |
| ZEB2 | **2** | - | - | - | - | n/a |
| ZNF507 | **4** | - | - | - | - | n/a |
| ZNF804A | **7** | **2** | - | - | **1** | n/a |
| ZNHIT6 | **2** | **1** | - | - | - | n/a |

***Legend****: Table summarizes the number of different variant types, detected in the investigated genes. Table only includes rare variants, as defined in the Methods sections. N/a = not applicable. Next generation sequencing is not suitable for detection of repeat expansion events. Repeat expansions were only investigated in the FMR1 gene, in order to diagnose Fragile-X patients.*
